# Trigeminal pain is suppressed by non-invasive vagal nerve stimulation in a rat headache model

**DOI:** 10.1186/1129-2377-14-S1-P80

**Published:** 2013-02-21

**Authors:** ML Oshinsky, AL Murphy, ME Cooper, BJ Simon

**Affiliations:** 1 Thomas Jefferson University, USA; 2 Thomas Jefferson Unversity, USA; 3 ElectroCore LLC., USA

## Introduction

Non-invasive vagal nerve stimulation (nVNS) is a novel technology for painless stimulation of the vagus nerve through the skin on the neck. Using a rat model of trigeminal allodynia, we tested the ability of nVNS to suppress the behavioral response and neurotransmitter changes following trigeminal pain. In this model, nociceptor activation is induced by infusing an inflammatory soup onto the dura in awake rats. This is repeated 3 times per week for 4 weeks. This stimulation leads to a state of secondary chronic pain on the face, which matches the pattern of referred pain in the migraineur.

## Objective

To study the mechanism of action of nVNS on behavioral and physiological correlates of trigeminal pain.

## Methods

One week after the last infusion, electrodes were positioned on the neck over the vagus nerve and the animal was stimulated for 1min. The nVNS signal consisted of 1ms bursts of a 5kHz sine wave repeated at 25Hz. The peak voltage applied was 22V. The control for nVNS was electrode placement, without stimulation. Another group of rats were used for microdialysis studies to determine the mechanism of action of nVNS effect on trigeminal pain. Rats were anesthetized and placed in a stereotaxic frame. Using a small microdialysis probe, extracellular amino acids were sampled for up to 3.5hr after GTN (0.1mg/kg) treatment with and without nVNS stimulation.

## Results

Following the 10 infusions of the IS, the rats were allodynic. All of the rats responded to nVNS stimulation with an increase in their periorbital pain threshold within 5min. This effect was maintained for at least 3.5hr after the stimulator was turned off. GTN treatment elicited a >7 fold increase in extracellular glutamate, which peaked at ~2hr after treatment. This increase in glutamate was completely blocked by 2min of nVNS stimulation.

## Conclusion

In a rat model of recurrent headache, both behavioral and physiological measures of trigeminal pain and allodynia are suppressed by 1-2min of nVNS. Funding: NIH R01-NS061571, ElectroCore LLC. COI: Bruce Simon is an employee of ElectroCore LLC.
